# Metabolism of the Endocannabinoid Anandamide: Open Questions after 25 Years

**DOI:** 10.3389/fnmol.2017.00166

**Published:** 2017-05-29

**Authors:** Mauro Maccarrone

**Affiliations:** ^1^Department of Medicine, Campus Bio-Medico University of RomeRome, Italy; ^2^European Center for Brain Research, IRCCS Santa Lucia FoundationRome, Italy

**Keywords:** biosynthesis, hydrolysis, signal transduction, storage, trafficking, transport

## Abstract

Cannabis extracts have been used for centuries, but its main active principle ∆^9^-tetrahydrocannabinol (THC) was identified about 50 years ago. Yet, it is only 25 years ago that the first endogenous ligand of the same receptors engaged by the cannabis agents was discovered. This “endocannabinoid (eCB)” was identified as *N*-arachidonoylethanolamine (or anandamide (AEA)), and was shown to have several receptors, metabolic enzymes and transporters that altogether drive its biological activity. Here I report on the latest advances about AEA metabolism, with the aim of focusing open questions still awaiting an answer for a deeper understanding of AEA activity, and for translating AEA-based drugs into novel therapeutics for human diseases.

## From Phytocannabinoids to Endocannabinoids

Cannabis (*Cannabis sativa* or* Cannabis indica*) extracts have been used in folklore medicine for centuries, and at present the potential benefits and harms to human health of legalizing the therapeutic and/or recreational cannabis use are a major issue for public authorities and opinion leaders worldwide. It took almost 30 years after the isolation of the psychoactive ingredient of cannabis, ∆^9^-tetrahydrocannabinol (THC) in 1964 (Gaoni and Mechoulam, 1964) to discover the first endogenous lipophilic molecule able to activate the same G protein-coupled type-1 (CB_1_) and type-2 (CB_2_) cannabinoid receptors activated by THC (Pertwee et al., 2010). In 1992 this “endocannabinoid (eCB)” was found to be *N*-arachidonoylethanolamine, also known as anandamide (AEA), the “amide of the inner bliss = ananda” (Devane et al., 1992). Shortly after the identification of AEA, another major eCB was discovered, 2-arachidonoylglycerol (Mechoulam et al., 1995; Sugiura et al., 1995), and both compounds are still recognized as the two main members of an ever-growing family of bioactive lipids (Mechoulam et al., 2014). Indeed, other important ω-6 (*n*-6) fatty acid compounds with cannabimimetic properties, such as *N*-arachidonoyldopamine, 2-arachidonoylglycerylether (noladin ether) and *O-arachidonoylethanolamine* (virodhamine), have been listed among eCBs (Fezza et al., 2014), along with ω-3 (*n*-3) fatty acid derivatives like *N*-docosahexaenoylethanolamine (Brown et al., 2010). Additionally, “eCB-like” compounds such as *N-palmitoylethanolamine*, *N*-oleoylethanolamine, and *N*-stearoylethanolamine have been shown to exert a CB_1_CB_2_-independent “entourage effect” that potentiates the activity of eCBs at their receptor targets (Ben-Shabat et al., 1998). More recently, two novel “eCB-like” compounds derived from juniperonic acid, the ω-3 structural isomer of arachidonic acid (AA), were identified in the plant kingdom, suggesting that distinct *N*-acylethanolamines may occur in different monophyletic taxa (Gachet et al., 2017).

In the last 25 years, AEA and congeners have been shown to play key biological activities, in both the central nervous system (Maccarrone et al., 2014; Di Marzo et al., 2015; Soltesz et al., 2015; Curran et al., 2016), and the periphery (Maccarrone et al., 2015; Benyó et al., 2016; Jourdan et al., 2016; Sharkey and Wiley, 2016; Wang et al., 2016). Such a multifaceted ability of AEA to impact on virtually every system of human body (and well beyond humans along the phylogenetic tree) depends on a multiplicity of receptor targets that include, besides CB_1_ and CB_2_, transient receptor potential vanilloid-1 (TRPV1) channels, G-protein coupled receptors 55 (GPR55) and 119 (GPR119), and peroxisome proliferator activated receptors (PPARs; reviewed in Maccarrone et al., 2014, 2015; Di Marzo et al., 2015; Soltesz et al., 2015; Benyó et al., 2016; Curran et al., 2016; Jourdan et al., 2016; Sharkey and Wiley, 2016; Wang et al., 2016). These receptor-mediated activities of AEA, the underlying signal transduction pathways and the related target diseases, will not be covered in the present review, which focuses on metabolism, storage and trafficking that control endogenous content, and thence biological activity, of AEA. The aim is to put in a better perspective open questions that remain to be answered for a deeper understanding of AEA activity, and for the possible translation of AEA-based drugs into novel therapeutics for human diseases.

## Metabolism of AEA

**Figure 1:**
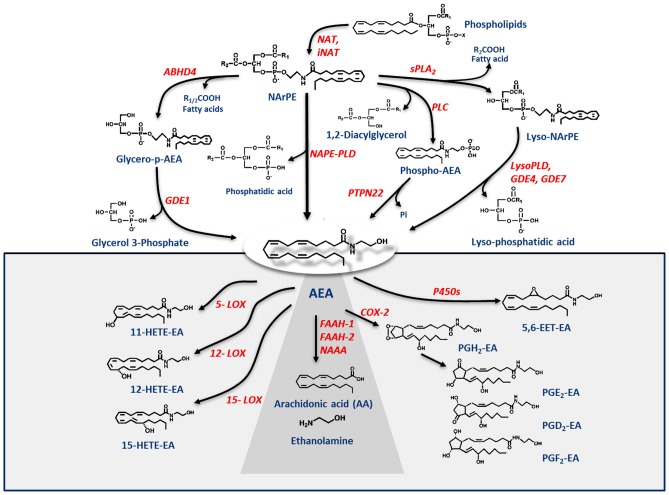
Metabolic pathways of AEA synthesis, degradation and oxidation. See text for details. Abbreviations: AEA, *N*-arachidonoylethanolamine (anandamide); NAT, *N*-acyltransferase; NAPE, *N*-acyl-phosphatidylethanolamine; NAPE-PLD, NAPE-specific phospholipase D; NArPE, *N*-arachidonoyl-phosphatidylethanolamine; ABHD4, α/β-hydrolase domain 4; GDE, glycerophosphodiesterase; PLC, phospholipase C; PTPN22, non-receptor protein tyrosine phosphatase 22; sPLA_2_, soluble phospholipase A_2_; FAAH, fatty acid amide hydrolase; NAAA, *N*-acylethanolamine-hydrolyzing acid amidase; COX-2, cyclooxygenase-2; LOX, lipoxygenase; P450s, cytochrome P450 monooxygenases; PG-EA, prostaglandin-ethanolamide; HETE-EA, hydroxyeicosatetraenoyl-ethanolamide (hydroxy-AEA); EET-EA, epoxyeicosatrienoyl-ethanolamide.

Shortly after the discovery of AEA, it was found that its biosynthesis occurs by release from membrane phospholipid precursors (Di Marzo et al., 1994; Cadas et al., 1997). A striking feature that emerged in the following years was that AEA biosynthetic pathways are apparently redundant (for reviews see Ueda et al., 2013; Fezza et al., 2014; Cascio and Marini, 2015; Battista and Maccarrone, 2017). The best characterized enzymes that synthesize AEA are shown in Table 1, and their reactions are schematically depicted in Figure 1. Among these routes, the sequential action of a Ca^2+^-dependent (Cadas et al., 1997; Ogura et al., 2016) or independent *N*-acyltransferase (NAT or iNAT, respectively; Jin et al., 2007, 2009), and then of *N*-acyl-phosphatidylethanolamine (NAPE)-specific phospholipase D (NAPE-PLD; Okamoto et al., 2004) appears the most relevant biosynthetic pathway of AEA.

**Table 1:** Main biosynthetic, hydrolytic and oxidative enzymes of N-arachidonoylethanolamine (or anandamide (AEA)).

Name (abbreviation)	Molecular mass	Intracellular localization	E.C. number
Ca^2+^-dependent *N*-acyltransferase (NAT)	Unknown	Integral membranes	2.3.1.x
Ca^2+^-independent *N*-acyltransferase (iNAT)	Unknown	Mainly cytoplasm	2.3.1.x
*N*-Acyl-phosphatidylethanolamine-specific phospholipase D (NAPE-PLD)	46 kDa	Associated to membranes	3.1.4.4
α/β-Hydrolase domain 4 (ABHD4)	39 kDa	Associated to membranes	3.1.1.x
Protein tyrosine phosphatase, non-receptor type 22 (PTPN22)	91 kDa	Mainly cytoplasm	3.1.3.48
Secretory phospholipase A_2_ (sPLA_2_)	16 kDa	Membrane caveolae and perinuclear sites	3.1.1.4
Lysophospholipase D (LysoPLD)	101 kDa	-	3.1.4.4
Glycerophosphodiesterase 1 (GDE1)	37 kDa	Membranes	3.1.4.46
Glycerophosphodiesterase 4 (GDE4)	36 kDa	Membranes and cytoplasm, especially at cell periphery and perinuclear sites	3.1.4.46
Glycerophosphodiesterase 7 (GDE7)	37 kDa	Cytoplasm and partially endoplasmic reticulum	3.1.4.46
Fatty acid amide hydrolase-1 (FAAH-1)	63 kDa	Associated to membranes (mainly in the endoplasmic reticulum), adiposomes	3.5.1.99
Fatty acid amide hydrolase-2 (FAAH-2)	58 kDa	Associated to membranes, adiposomes	3.5.1.99
*N*-Acylethanolamine-hydrolyzing acid amidase (NAAA)	31 kDa	Mainly in lysosomes	3.5.1.4
5-Lipoxygenase (5-LOX)	78 kDa	Cytoplasm, adiposomes	1.13.11.34
12-Lipoxygenase (12-LOX)	76 kDa	Cytoplasm, adiposomes	1.13.11.31
15-Lipoxygenase (15-LOX)	75 kDa	Cytoplasm, adiposomes	1.13.11.33
Cyclooxygenase-2 (COX-2)	69 kDa	Mitochondria, adiposomes	1.14.99.1
Cytochromes P450 (P450s)
CytP4504F2	60 kDa	Microsomes, mitochondria	1.14.13.30
CytP4503A4	57 kDa		1.14.13.97
CytP4X1	59 kDa		1.14.14.1

Degradation of AEA into AA and ethanolamine also depends on multiple enzymes. Shortly after the discovery of the first AEA amidase activity in different cell types (Koutek et al., 1994; Hillard et al., 1995), it was found that fatty acid amide hydrolase (FAAH) is the main responsible for AEA cleavage (Cravatt et al., 1996; Giang and Cravatt, 1997). Cloning, crystal structure, kinetic properties and body distribution of FAAH have been extensively reviewed (Cravatt and Lichtman, 2003; McKinney and Cravatt, 2005), and *faah* knockout mice have definitively confirmed its pivotal role in controlling AEA tone *in vivo*, because these animals have ~15-fold higher levels of AEA than wild-types (Cravatt et al., 2001, 2004). The best characterized enzymes that degrade AEA are shown in Table 1, and their reactions are schematically depicted in Figure 1 (see Fezza et al., 2014; and Battista and Maccarrone, 2017; for extensive reviews).

In addition to hydrolytic pathways, AEA can be subjected to oxygenation by cyclooxygenase-2 (COX-2; Kozak et al., 2001, 2002; Rouzer and Marnett, 2011; Hermanson et al., 2013), 5-,12- and 15-lipoxygenase (5-/12-/15-LOX; Hampson et al., 1995; Edgemond et al., 1998; Van der Stelt et al., 2000), as well as by several cytochrome P450 monooxygenases (P450s; Snider et al., 2010; Urquhart et al., 2015), as shown in Table 1. COX-2 turns AEA into prostaglandin-ethanolamides (PGs-EA), while LOXs convert it into hydroxy-anandamides or hydroxyeicosatetraenoyl-ethanolamides (HETEs-EA), and P450s into epoxyeicosatrienoyl-ethanolamides (EETs-EA), as schematically depicted in Figure 1. Accumulated evidence suggests that oxygenated derivatives of AEA have a biological activity of their own, but their impact on human health and disease remains to be clarified. Incidentally, it should be noted that recent data suggest that *in vivo* COX-2 might even prefer eCBs over AA as natural substrates (Hermanson et al., 2013), widening the spectrum of lipid signals that can be affected by COX-2 inhibitors like nonsteroidal anti-inflammatory drugs (Hermanson et al., 2014). Therefore, it seems urgent to further interrogate the impact of these large and widely consumed therapeutic drugs on eCB metabolism in pathophysiological processes of our body.

## Storage and Trafficking of AEA

The classical “dogma” that AEA is synthesized and released on demand via hydrolysis of cell membrane phospholipid precursors has been recently revisited (Maccarrone et al., 2010; Min et al., 2010), also on the basis of unexpected evidence for intracellular reservoirs and transporters of eCBs. These new entities have been shown to drive intracellular trafficking of eCBs, thus adding a new dimension to the regulation of their biological activity (Maccarrone et al., 2010).

Storage of AEA has been documented in adiposomes (lipid droplets), that constitute a dynamic reservoir for the sequestration of this eCB (Oddi et al., 2008). Remarkably, confocal microscopy and biochemical studies revealed that also FAAH-1 (Oddi et al., 2008) and FAAH-2 (Kaczocha et al., 2010) are spatially associated with lipid droplets, and that cells with a larger adiposome compartment have enhanced AEA catabolism by both enzymes. FAAH-2 displays also a putative N-terminal hydrophobic region as a functional lipid droplet localization sequence (Kaczocha et al., 2010). Overall, these findings suggest that adiposomes may have a critical role in accumulating AEA, and possibly in connecting plasma membrane to internal organelles along the metabolic route of this eCB. In line with these data, depletion of a pre-existing pool of 2-arachidonoylglycerol has been recently shown as a key event in sperm activation (Miller et al., 2016), speaking against the on demand synthesis of this eCB much alike that of AEA.

**Figure 2:**
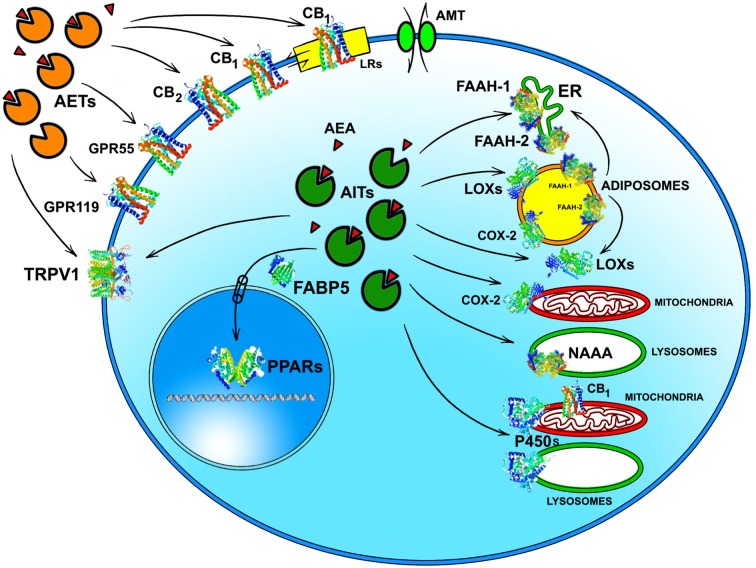
Intracellular and extracellular trafficking of AEA. See text for details. 3D structures were drawn by using the following source files: 5TGZ.pdb human (CB_1_); 5IRZ.pdb rattus norvegicus (TRPV1); 2I4J.pdb ppar-gamma human (PPARs); 4LKP.pdb apo human epidermal fatty acid binding protein (FABP5); 1MT5.pdb rattus norvegicus (FAAH-1); 4NRE.pdb human 15-lipoxygenase-2 (LOXs); 4RRW.pdb apo murine COX-2; 2JJN.pdb closed cytochrome P450 EriK (P450 s). Crystal structures are not yet available for CB_2_, GPR55 and GPR119, therefore to represent these G protein coupled receptors data of β1-adrenergic receptor (5A8E.pdb) were used. Finally, for FAAH-2 and NAAA the same 3D structure as FAAH-1 was used, in the absence of crystallographic data. All 3D structures were drawn by means of the Jsmol software, a JavaScript framework (open source) available at www.RCSB.org. Abbreviations: CB_1_, type-1 cannabinoid receptor; CB_2_, type-2 cannabinoid receptor; AEA, *N*-arachidonoylethanolamine (anandamide); TRPV1, transient receptor potential vanilloid-1; GPR55, G-protein coupled receptor 55; GPR119, G-protein coupled receptor 119; PPARs, peroxisome proliferator activated receptors; FAAH, fatty 523 acid amide hydrolase; NAAA, *N*-acylethanolamine-hydrolyzing acid amidase; COX-2, cyclooxygenase-2; LOXs, lipoxygenases; P450s, cytochrome P450 monooxygenases; AET, AEA extracellular transporter; AITs, intracellular AEA transporters; AMT, AEA membrane transporter; ER, endoplasmic reticulum; FABP5, fatty acid binding protein 5; LRs, lipid rafts.

Whatever the physiological relevance of AEA accumulation, the number of alternate targets that a single cell can have for this eCB poses the question of how AEA can reach the right target in a timely manner and at a suitable concentration for effective action. It should be recalled that the lipid nature of AEA hampers its free movement in aqueous mediums like cytosol. Thus, intracellular AEA transporters (AITs) should exist that ferry AEA to the correct final destination, like: (i) endoplasmic reticulum (ER) for degradation by FAAH-1/FAAH-2; (ii) adiposomes for accumulation, degradation by FAAH-1/-2 or oxidation by COX-2 or LOXs; (iii) mitochondria for oxidation by COX-2 or P450 s, and possible activation of CB_1_ (Bénard et al., 2012); (iv) lysosomes for degradation by P450s or NAAA; or (v) nucleus for activation of PPARs (Figure 2). Interestingly, AITs have been indeed found in different cell types, and include fatty acid binding proteins (FABPs; Kaczocha et al., 2009), heat shock protein 70 (HSP70) and albumin (Oddi et al., 2009), FAAH-1-like AEA transporter (FLAT-1; Fu et al., 2011), and potentially sterol carrier protein 2 (SCP-2; Liedhegner et al., 2014). Of note, the role of a specific AIT that may deliver AEA where and when needed has been recently demonstrated in a study showing the ability of FABP5 to drive AEA to nuclear PPARs (Kaczocha et al., 2012).

Much alike intracellular trafficking, cell-to-cell transport of AEA requires AEA extracellular transporters (AETs), as depicted in Figure 2. The identity of such AETs remains to be elucidated, but recent data point to microvesicles as good candidates (Gabrielli et al., 2015). Also FABP4 (Hotamisligil and Bernlohr, 2015), albumin and HSP70 (Shevtsov and Multhoff, 2016) are known to be secreted extracellularly, thus it is conceivable that they play a role as AETs. Yet, their actual contribution to AEA extracellular transport remains to be demonstrated. Noteworthy AETs might drive AEA to CB_1_ receptors localized within or outside cholesterol-enriched membrane microdomains like lipid rafts (LRs), thus modulating receptor activation. Indeed, it has been shown that cholesterol present in LRs reduces AEA binding to CB_1,_ and hence signal transduction triggered thereof (Oddi et al., 2012).

Finally, transport of AEA across the plasma membrane has been highly debated over the last 20 years, with a number of studies pointing towards the existence of a true AEA membrane transporter (AMT), but as many pointing against it (see Fowler, 2013; Nicolussi and Gertsch, 2015; Deutsch, 2016). While the discussion of evidences in favor or against AMT goes beyond the scope of this review, is worthwhile to stress that a saturable uptake of AEA can certainly be due to a facilitated transport or to a passive diffusion driven by intracellular hydrolysis, most likely by FAAH-1. Yet, a saturable export of AEA, that has been reported shortly after the discovery of this eCB (Hillard et al., 1997), and then by several independent studies (see Chicca et al., 2012, and references therein), cannot depend on a concentration gradient driven by intracellular hydrolysis. Therefore, a clear evidence of a saturable bidirectional transport of AEA appears to strongly speak in favor of the existence of a true AMT. A reason for the still missing molecular identity of an AMT could be that it is a multimeric protein rather than a monomer, making it difficult to identify and isolate all subunits and reconstitute a functional AMT from them. Preliminary evidence for the assembly of an AEA uptake machinery within caveolae/lipid rafts has been indeed reported (McFarland et al., 2004), though its composition has not yet been determined.

## Open Questions and Future Directions

The previous sections have shown a rather complex AEA metabolism, storage and trafficking, strongly suggesting that for every cell it is important to properly synthesize, degrade and transport this eCB. In fact the multiplicity of pathways that lead to AEA release from membrane phospholipid precursors, and then to its cleavage (Table 1; Figure 1), should have not passed natural selection unless they might have conferred an advantage for survival. It is apparent that FAAH-1 is a key controller of AEA tone *in vivo*, and therefore many inhibitors have been developed over the last decade to block its activity, thus enhancing content and biological activity of AEA. Despite the potential of these compounds as innovative therapeutics (reviewed by Bisogno and Maccarrone, 2013; Fowler, 2015), better drugs are still needed for effective cure or slowing down of human pathologies. In this context, it seems noteworthy that subtle differences exist between rodent and human FAAH-1 that impact on the efficacy of inhibitors, thus leading to rodent/human ratios of IC_50_ values from ~0.3 to ~4.0 (Di Venere et al., 2012). The consequences on translation of preclinical studies to the patient’s bedside are apparent. Moreover, the selectivity of an inhibitor of FAAH-1 should be carefully checked towards effects on other components of the so-called “endocannabinoid system (ECS)” which includes different metabolic enzymes and carriers of AEA, in addition to the proteins that bind, metabolize and transport 2-arachidonoylglycerol (Maccarrone et al., 2014). Thus, a valuable FAAH-1 inhibitor should show at least very little (if any) effects on other ECS proteins, as well as potential non-ECS off-targets. Yet it is not always obvious that these tests are performed during new drug development programs (Di Venere et al., 2012). In this context, a dramatic outcome of a recent phase I clinical trial with the purported FAAH-1 inhibitor BIA 10-2474 led to an unanticipated severe toxic cerebral syndrome whose underlying mechanisms remain unknown (Kerbrat et al., 2016). This adverse event clearly demonstrates the importance of a thorough characterization of the specificity of any new FAAH-1-oriented drug. Indeed, conclusions of an *ad hoc* temporary specialized scientific committee and the fact that phenomena resembling those seen in humans with BIA 10-2474 have not been reported in the literature in any of the numerous animal studies and clinical trials with various inhibitors of FAAH (e.g., PF-04457845, JNJ-42165279, SSR-411298, V-158866 and URB597, just to list those with more advanced programs), strongly (yet not conclusively) suggest that off-target effects of BIA 10-2474 itself or a metabolite thereof, and not FAAH-1 inhibition was the cause of adverse reactions (Mallet et al., 2016; Edan and Kerbrat, 2017). Moreover, it should be recalled that unexpected regulators of FAAH-1 activity are emerging, such as membrane cholesterol that favors the access of AEA to the enzyme active site (Dainese et al., 2014). Thus it remains to be clarified to what extent the lipid environment may tune FAAH-1 activity *in vivo*, and how it can be exploited to design more effective FAAH-1 inhibitors.

Unlike degradation, the key enzymes for AEA biosynthesis are still to be identified, though NAPE-PLD seems to play a pivotal role. Anyhow selective inhibitors of AEA biosynthetic enzymes are not yet available, making it difficult to dissect the contribution of each pathway to the overall synthesis of this eCB. In addition, as shown in knockout animals the lack of an enzyme can be compensated by an alternate route. Therefore, development of effective inhibitors of distinct AEA biosynthetic enzymes, and of conditional knockout mice where a specific enzyme can be switched off at will, is deemed necessary to boost our understanding of AEA biosynthesis. Better methods and more accurate measurements are required to this aim. This has been indeed the case for FAAH-1, whereby activity-based protein profiling has allowed to identify truly selective blockers with low or no activity towards off-targets (Simon and Cravatt, 2010). Additionally, the quest for 3D structures of AEA metabolic enzymes should be actively pursued, because this type of information can certainly favor drug development. To date, a 3D crystal structure has been obtained only for FAAH-1 (Bracey et al., 2002), for which unsurprisingly different classes of effective inhibitors have been developed and investigated in detail (Table 2).

**Table 2:** Main classes of human fatty acid amide hydrolase-1 (FAAH-1) inhibitors.

Class	IC_50_ value	Patent (year)
**Carbamates**
URB-597	5 nM	US2004127518 (2004)
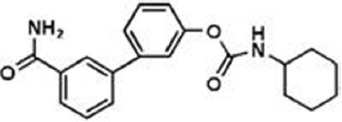
URB-937 (peripherally restricted)	27 nM	WO2012015704 (2012)
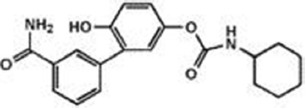
**Ethylaminopyrimidines**
JNJ-40413269	5 nM	WO2009105220 (2009)
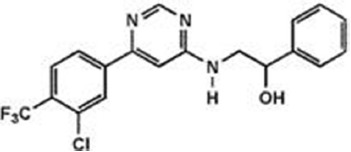
**Tetrahydronaphthyridines**		
RN-450	1 nM	WO2009011904 (2009)
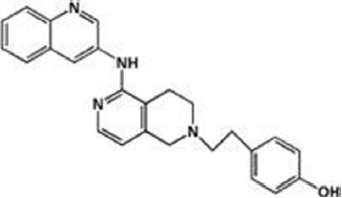
**Aryl ureas**
PF-04457845	7 nM	WO2009127944 (2009)
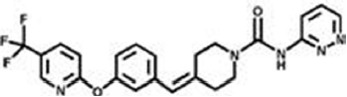
**Imidazoles**
MK-3168	1 nM	WO2010101724 (2010)
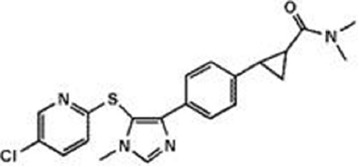
**α-Ketoheterocycles**
OL-135	2 nM	WO2010005572 (2010)
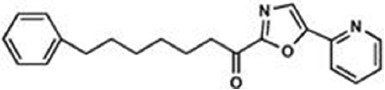

Of basic importance appears to be the identification and characterization of *in vivo* activity of distinct AITs and AETs that may drive AEA to the right target within and outside the cell (Figure 2). Each AIT and/or AET could be a further regulatory player within the cell, adding a new dimension to the already complex AEA-dependent signaling thus representing a target for novel AEA-based drugs.

Finally, genetic manipulation of the *faah-1* gene allowed to generate not only *faah-1* knockout (Cravatt et al., 2001) but also *faah-1* knockdown animals, where this AEA hydrolase has been deleted from peripheral tissues only (Cravatt et al., 2004). Studies performed on these allelic variants highlighted a different susceptibility to drug/alcohol abuse (Sipe et al., 2002; Chiang et al., 2004; Zhou et al., 2016), supporting a potential link between functional abnormalities in eCB signaling and drug/alcohol dependance. This is just an example of the potential impact of genetic manipulation of eCB system on human health (Zimmer, 2015), for instance on the widespread cannabis use disorder (Boileau et al., 2016). In addition, accumulating evidence points to AEA as a unique natural repressor of gene transcription, via epigenetic mechanisms that include increased DNA methylation (Paradisi et al., 2008), reduced histone acetylation and microRNA (D’Addario et al., 2013). It should be recalled that DNA methylation is a fundamental epigenetic modification of the genome that is involved in a large number of cellular processes, like embryonic development, transcription, chromatin structure, X chromosome inactivation, and genomic imprinting and chromosome stability. Among many other diseases, a role for altered methylation has been established in cancer, for which DNA hypomethylation is a hallmark (Paradisi et al., 2008). Against this background, the potential of AEA as a natural anti-cancer agent appears very promising, and certainly worth of urgent investigations.

## Conclusions

After 25 years much has been learned about AEA metabolism and transport, and this knowledge may serve as a paradigm to appreciate the complex machinery that can regulate many other lipid signals. Yet, much remains to be clarified in the next future, in order to better understand AEA signaling regulation and then translate basic AEA research into AEA-based therapeutics. In particular, it seems challenging that AEA and its metabolic enzymes hold potential also as much desired peripheral (blood) biomarkers of human diseases affecting tissues not easy to reach. For instance, FAAH-1 activity and expression are up-regulated in Alzheimer’s disease patients (D’Addario et al., 2012), unlike any other ECS element analyzed (including CB_1_, CB_2_, and NAPE-PLD). Moreover, AEA content, NAPE-PLD and FAAH-1 (reviewed by Rapino et al., 2014), and more recently also FAAH-2 (Tedeschi et al., 2017), have been shown to undergo distinct changes in distinct human reproductive disorders. In the new era of nanoscopy, it can be anticipated that also visualization of AEA and its metabolic machinery will greatly increase our understanding of signal transduction pathways triggered by this eCB. Availability of new tools like biotin-AEA (Fezza et al., 2008), positron-emission tomography (PET) probes (Boileau et al., 2016), and luciferin nanoparticles (Yuan et al., 2016) for FAAH-1 visualization, as well as of substrates for *in vivo* bioluminescence detection of FAAH-1 enzymatic activity (Mofford et al., 2015), indicates that the way to accurate location of distinct elements of AEA signaling within the cell has been already paved.

## Author Contributions

MM wrote the manuscript.

## Conflict of Interest Statement

The author declares that the research was conducted in the absence of any commercial or financial relationships that could be construed as a potential conflict of interest.
